# Control of DNA minor groove width and Fis protein binding by the purine 2-amino group

**DOI:** 10.1093/nar/gkt357

**Published:** 2013-05-09

**Authors:** Stephen P. Hancock, Tahereh Ghane, Duilio Cascio, Remo Rohs, Rosa Di Felice, Reid C. Johnson

**Affiliations:** ^1^Department of Biological Chemistry, David Geffen School of Medicine at the University of California at Los Angeles, Los Angeles, CA 90095-1737, USA, ^2^Center S3, CNR Institute of Nanoscience, Via Campi 213/A, 41125 Modena, Italy,^3^Department of Energy Institute of Genomics and Proteomics, University of California at Los Angeles, Los Angeles, CA 90095-1570, USA, ^4^Molecular and Computational Biology Program, Department of Biological Sciences, ^5^Department of Chemistry, ^6^Department of Physics and Astronomy, ^7^Department of Computer Science, University of Southern California, Los Angeles, CA 90089, USA and ^8^Molecular Biology Institute, University of California at Los Angeles, Los Angeles, CA 90095, USA

## Abstract

The width of the DNA minor groove varies with sequence and can be a major determinant of DNA shape recognition by proteins. For example, the minor groove within the center of the Fis–DNA complex narrows to about half the mean minor groove width of canonical B-form DNA to fit onto the protein surface. G/C base pairs within this segment, which is not contacted by the Fis protein, reduce binding affinities up to 2000-fold over A/T-rich sequences. We show here through multiple X-ray structures and binding properties of Fis–DNA complexes containing base analogs that the 2-amino group on guanine is the primary molecular determinant controlling minor groove widths. Molecular dynamics simulations of free-DNA targets with canonical and modified bases further demonstrate that sequence-dependent narrowing of minor groove widths is modulated almost entirely by the presence of purine 2-amino groups. We also provide evidence that protein-mediated phosphate neutralization facilitates minor groove compression and is particularly important for binding to non-optimally shaped DNA duplexes.

## INTRODUCTION

Protein binding to DNA is central to all aspects of chromosome function. Proteins can bind DNA over a range of affinities and specificities that are fine-tuned through multiple mechanisms depending on the individual protein and its function at particular binding sites. Binding site selection is governed by both direct readout of the chemical features of DNA bases (‘direct readout’) (1,2) and by indirect readout of sequence-dependent DNA conformation or deformability, also known as ‘shape readout’ (3,4). Direct contacts can be readily observed in high-resolution structures of protein–DNA complexes, but features of DNA shape readout have been more difficult to elucidate. Nevertheless, shape recognition can contribute in essential ways to DNA site selection by DNA-binding proteins that display both stringent and relaxed sequence specificity (4,5).

An important subset of sequence-dependent DNA conformation is the shape of the minor groove. Variability in minor groove geometry was recognized as soon as the first B-DNA crystal structures were determined (6), and its importance for protein recognition has recently gained renewed appreciation. For example, DNA segments with subtle variations in minor groove widths can direct Hox proteins to their different target sites with exquisite specificity (7). In these and other examples, basic side chain residues, most often arginines, selectively insert into the minor grooves because of shape-dependent differences in electrostatic potential (8). Similar principals contribute to the phasing of nucleosomes and selective binding of abundant nucleoid-associated proteins in bacteria (8–12).

Variation in minor groove widths can be the critical determinant mediating overall shape complementarity between the DNA ligand and protein surface (13). For example, expanded minor grooves are required for formation of DNA complexes with HMGB and PurR/LacI repressors where α-helices are inserted into the groove (14–16). On the other hand, compressed minor grooves within the center of the binding site are essential for regulatory proteins like phages 434 and P22 c2 repressors, the papillomavirus E2 transactivator and the Fis nucleoid-associated protein to fit into adjacent major groove surfaces (17–20).

**Figure 1.**
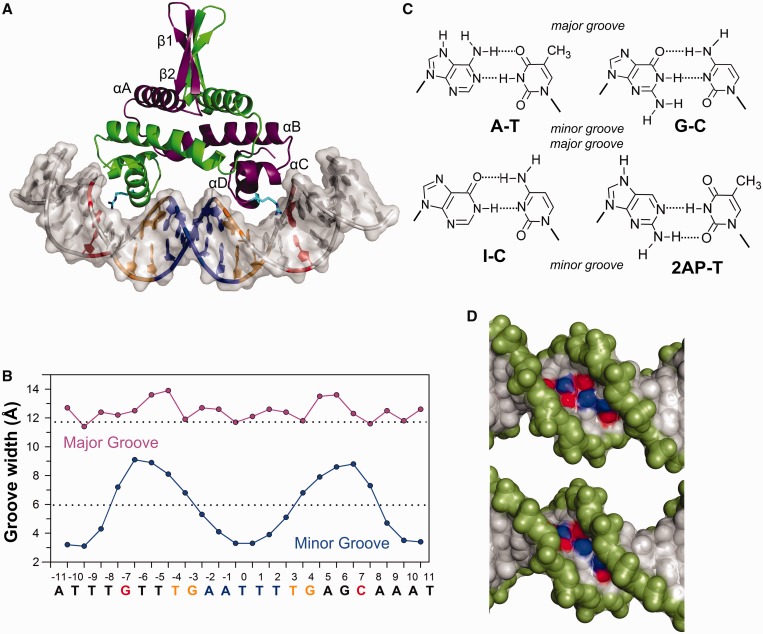
DNA groove widths in the Fis–DNA complex. (**A**) Crystal structure of Fis bound to the high-affinity binding site F1 (PDB ID: 3IV5). Fis binds DNA as a homodimer (green and purple subunits) and inserts two recognition α-helices (αD) into consecutive major grooves of the target DNA site. The Arg85 side chain, which mediates the only important base contact to the conserved guanines near the outer edges of the binding site, is highlighted in cyan. (**B**) Major (magenta) and minor (blue) groove widths are plotted over the length of the Fis-bound DNA. Values represent the distance between closest interstrand phosphates minus their van der Waals radii. Dashed lines represent canonical groove widths for B-DNA. The sequence of the F1 site is on the bottom and is color-coded in-line with A. (**C**) Chemical structures of DNA base pairs used in this study. (**D**) Space-filling view looking into the minor groove at the center of the F28 (GCG) sequence (blue: N2 atoms, red: O2 atoms). Top is DNA constructed *in silico* using mean free-DNA parameters (minor groove width at center = 7.1 Å); bottom is the DNA within the Fis complex (minor groove width at center = 4.4 Å).

Fis, the model system used in the present study, is one of the most abundant DNA-binding proteins in rapidly dividing enteric bacteria. It regulates a diversity of transcription and recombination reactions (21–24), and its DNA bending and looping activities implicate a role for Fis in the organization, compaction and dynamics of the bacterial chromosome (25). Stable Fis-binding sites share a highly degenerate 15 bp consensus motif consisting of G/C-13 bp-C/G where the central 5–7 bp are A/T rich (21,26–29). Recent Fis–DNA X-ray structures have revealed that the minor groove over the central 5 bp, as well as in the regions flanking the 15-bp motif, is compressed to a width that is about half of that observed for canonical B-form DNA, yet widths across the major groove vary only slightly (Figure 1A and B) (19). Compression of the central minor groove is essential for binding because of the unusually close spacing between recognition helices of each helix-turn-helix (HTH) motif of the dimer (30,31). All direct contacts between Fis and DNA are mediated within the adjacent major groove interfaces; most of these are with the DNA backbone with the only important base-specific contact being to a guanine at the edges of the 15-bp motif (Figure 1A) (19).

In general, crystal structures of free and protein-bound DNA show that A/T-rich sequences, especially A-tracts, contain narrow minor grooves, whereas more G/C-rich sequences have wider minor grooves (8,32–34); however, there are exceptions [e.g. (35,36)]. A distinguishing feature of G/C versus A/T base pairs within the minor groove is the exocyclic 2-amino group of guanine, which resides on the floor of the minor groove and donates a hydrogen to form a third Watson–Crick hydrogen bond with the 2-keto of cytosine (Figure 1C and D). There are numerous reports of the guanine 2-amino group influencing global DNA structure and flexibility and affecting interactions with water molecules, cations, small molecules and proteins (37–42). However, high-resolution X-ray crystal structures comparing DNA duplexes in which guanines are replaced with inosines that lack the 2-amino group (Figure 1C) show remarkably subtle atomic differences (43,44). Thus, we are without a clear structural picture of how the purine 2-amino group affects minor groove geometry.

Here, we investigate the role of the purine 2-amino group in controlling the shape of the minor groove by evaluating Fis-binding efficiency and DNA structures in Fis-bound complexes when guanine, inosine or 2-aminopurine (2AP) bases are substituted for adenine within the center of the binding site. These data, combined with molecular dynamics (MD) simulations of unbound Fis-binding sites with and without purine 2-amino groups, provide strong evidence that the presence of this exocyclic group is the primary DNA determinant that modulates intrinsic minor groove width. We also provide evidence that protein-induced neutralization of phosphates across the minor groove facilitates groove narrowing, particularly for DNA sequences that are suboptimal for compression.

## MATERIALS AND METHODS

### DNA binding and lifetime assays

Equilibrium binding was measured by electrophoretic gel mobility shift assays in the presence of 50 µg/ml poly dI/dC as described previously (19). Lifetime measurements were performed by adding ≥10 000-fold molar excess of unlabeled F1 duplex to preformed ^32^P-DNA–Fis complexes and following the time-dependent decay of the labeled complex by electrophoretic gel mobility shift assays (19,45). Fis protein for binding and crystallography studies was prepared as described previously (19). DNA for binding assays was obtained from Integrated DNA Technologies, Inc (IDT).

### Crystallization and structure determination

Fis and DNA were co-crystallized as described previously (19). DNA for crystallography was obtained from IDT, except for F28-2AP, which was from the Keck Oligonucleotide Synthesis Facility (Yale University) and gel purified. Data were collected at 100 K at the Advanced Photon Source (Chicago, IL, USA) beamline 24-ID-C (Table 1). Fis–DNA structures were solved by molecular replacement (PHASER) (46) using the Fis–F1 structure (PDB ID: 3IV5) as the search model. The models were refined using PHENIX (47,48). Examples of 2Fo-Fc maps showing electron densities over the DNA or selected base pairs are shown in Supplementary Figure S1. Data collection, refinement statistics and PDB codes are given in Table 1. DNA structure analysis and DNA modeling were performed using the 3DNA suite (49); mean values of sequence-specific base pairs and base pair steps were from Olson *et al.* (50). Structure figures were generated using PyMol (http://www.pymol.org).

**Table 1.** Crystallographic data collection and refinement statistics

Structure^a^	F28 (4IHV)	F28–dI (4IHW)	F28–2AP (4IHX)	F29–dI (4IHY)
Data collection				
Space group	P2_1_2_1_2_1_	P2_1_2_1_2_1_	P2_1_2_1_2_1_	P2_1_2_1_2_1_
Unit cell dimensions	a = 42.79	a = 43.37	a = 43.12	a = 43.21
	b = 89.39	b = 94.40	b = 91.59	b = 94.05
	c = 154.15	c = 154.99	c = 154.02	c = 155.04
Resolution range (Å)	90–2.7 (2.8–2.7)^b^	90–2.7 (2.8–2.7)	90–2.8 (2.9–2.8)	90–2.9 (3.0–2.9)
Completeness (%)	99.6 (97.5)	97.2 (95.8)	99.9 (99.9)	99.6 (99.9)
Redundancy	7.0 (6.8)	6.6 (6.5)	5.8 (6.0)	7.7 (7.5)
Rsym (%)	8.2 (88.0)	8.4 (84.8)	6.1 (95.4)	11.5 (94.7)
I/σI	23 (2.4)	14.4 (2.85)	16.45 (2.0)	19.0 (2.4)
Refinement				
Resolution (Å)	2.7	2.7	2.8	2.9
No. of reflections	16 603	17 708	15 566	14 611
R_work_	21.7	22.1	21.6	23.0
R_free_	25.7	26.0	25.9	27.4
RMSD bond length	0.008	0.010	0.010	0.008
RMSD bond angles	1.5	1.5	1.5	1.5
Number of atoms				
Protein	1505	1505	1505	1505
DNA	1101	1098	1101	1096
Water	5	2	0	0
B factors				
Protein	28.9	32.9	29.9	30.7
DNA	51.3	52.7	56.2	51.0
Water	23.4	22.9		
Ramachandran statistics				
Favored (%)	94.6	95.7	95.1	94.6
Allowed (%)	5.4	4.3	4.9	5.4
Generously allowed (%)	0	0	0	0

^a^PDB ID codes are given in parentheses.

^b^Highest resolution shell is given in parentheses.

### Molecular dynamics simulations

MD simulations were performed on DNA fragments in solution with the NAMD code (51) and the parmbsc0 AMBER force field (52). For inosine, which is not implemented in AMBER, we used *ab initio*-based charge parameters that were developed and tested elsewhere (53,54). The 15mers were extracted from the central portion of Fis-binding sequences F1, F28, F28–dI, F29 and F29–dI. The initial double helices were constructed using the 3DNA package with standard B-DNA parameters (49). Each oligomer was then solvated with explicit TIP3P (55) water molecules for a thickness of ∼16 Å from the DNA molecule in each direction. Na^+^ counterions were added in the simulation box for global system neutrality. The MD runs were conducted for 50 ns in the isothermal-isobaric (NPT) ensemble at room temperature and pressure (*T* = 300 K, P = 1.01325 bar), and the trajectories were collected every 1 ps. Groove widths for each snapshot were computed with Curves 5.3 (56) and analyzed for the last 40 ns of each 50 ns simulation.

The production MD run for each oligomer was preceded by a minimization-equilibration procedure, according to an established protocol (57). The minimization was based on the conjugate gradient algorithm. Each simulation (F1, F28, F28–dI, F29 and F29–dI) was articulated in different steps. (i) We initially performed solvent equilibration only: all the water molecules and cations were subjected to 20 ps dynamics, whereas the nucleic acid molecule was kept fixed with a SHAKE (58) tolerance of 10^−^^8^. During this solvent equilibration, the temperature of the solvent molecules was slowly raised to 100 K by coupling to the heat bath, and the pressure was kept fixed at 1.01325 bar using the Berendsen method (59). (ii) We performed a full-atom minimization of this partially equilibrated system. (iii) The quenched system was heated slowly from 0 to 300 K, by coupling it to a heat bath whose temperature was raised at the rate of 50 K every 10 ps. (iv) The system was equilibrated 100 ps longer at the temperature and pressure of 300 K and 1.01325 bar, respectively. (v) Finally, we ran the production MD run.

## RESULTS

### Crystal structure of Fis bound to DNA containing three central G/C base pairs reveals large changes in minor groove widths relative to A/T base pairs

**Figure 2.**
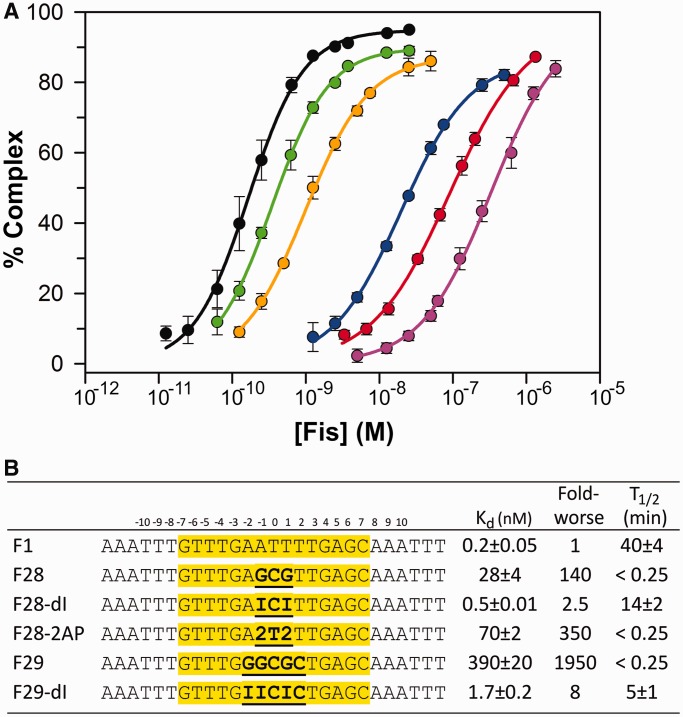
Sequences and Fis-binding properties of DNA duplexes used in this study. (**A**) Fis-binding isotherms to the different DNA substrates: F1 (black), F28 (blue), F28–dI (green), F28-2AP (red), F29 (magenta) and F29–dI (orange). (**B**) Sequences and summary of Fis-binding properties of the DNA substrates. I designates inosine, and 2 designates 2-amino purine (2-AP). Fold-worse is relative to F1.

The high-affinity Fis-binding sequence F1 contains an AT-rich center (AATTT) that is not contacted by the protein, but whose minor groove is severely compressed relative to average B-form DNA (Figure 1A and B). Substituting the three central base pairs of F1 with GCG (F28) reduces equilibrium Fis–DNA binding 140-fold and dramatically destabilizes the complex (Figure 2A and B).

**Figure 3.**
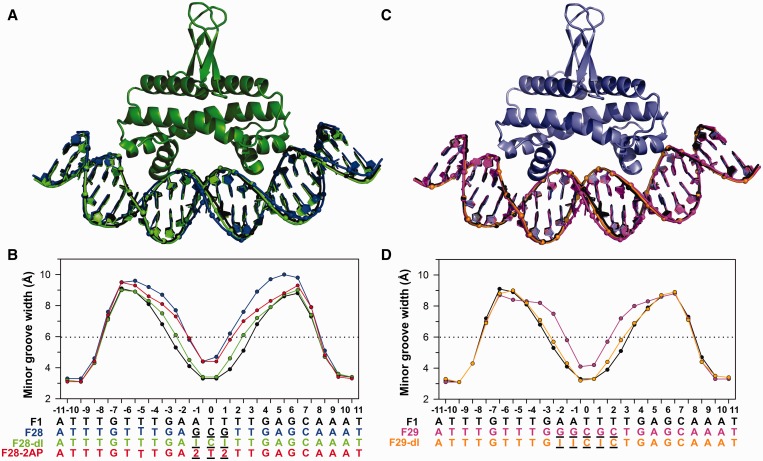
Minor groove widths within crystal structures of Fis–DNA complexes correlate with the presence of the purine 2-amino group. (**A**) Structures of Fis complexes with F28 DNA (blue), F28–dI DNA (green) and F1 DNA (black). Structures are aligned over the proteins, but only the protein of the F28–dI complex is shown. (**B**) Minor groove width plots of the DNA structures in (A) plus the F28–2AP (red) complex. (**C**) Structures of Fis complexes with F29 DNA (magenta, PDB ID: 3JRC), F29–dI DNA (orange) and F1 DNA (black). Structures are aligned over the proteins, but only the protein of the F29–dI complex is shown. (**D**) Minor groove width plots of the DNA structures in (C).

Although Fis binding to the F28 sequence is poor, we were able to obtain an X-ray crystal structure of the complex. The structure of the Fis–F28 complex was solved by molecular replacement using the Fis–F1 structure as a search model and was refined at 2.7 Å to an R_w_/R_f_ of 21.7/25.7 (Table 1). All of the Fis–DNA contacts present in the F1 structure are maintained in the F28 structure, and the protein backbone atoms of the F1 and F28 structures align with a root-mean-square deviation (RMSD) of 0.27 Å. The most striking difference between the F1 and F28 structures is the DNA minor groove width, which is significantly wider over the central portion of the protein–DNA interface in the F28 structure relative to the F1 structure (Figure 3A and B). The mean and minimum minor groove widths over the central 5 bp are increased by 1.8 Å and 1.1 Å, respectively, in the F28 structure relative to the F1 structure (Table 2). The minor groove widths at positions −4 to −7 and +4 to +7 are also expanded in the F28 complex relative to F1 (Figure 3A and B). Although the Fis–F28 structure contains only three G/C base pair substitutions, minor groove compression within the center of the core is compromised to a similar extent as in the previously determined Fis–F29 structure that contains five G/C base pair substitutions (Figure 3C and D) (19).

**Table 2.** Groove widths and mean DNA parameters over the central 5 bp of Fis–DNA complexes

−2 to +2 (5 bp)	F1	F28	F28–dI	F28–2AP	F29	F29–dI
Mean minor groove width (Å)	3.9	5.7	4.4	5.5	5.5	4.2
Minimum minor groove width (Å)	3.3	4.4	3.4	4.4	4.2	3.2
Mean helix twist (°)^a^	38	37	38	37	37	38
Mean roll (°)^a^	−2.0	−0.02	−2.5	−0.9	−1.0	−3.1
Mean propeller twist (°)^a^	−15.1	−9.7	−10.4	−13.2	−9.0	−10.0

^a^See Supplementary Figure S2 for plots of helix twist, roll and propeller twist.

### Removal of the purine 2-amino group enhances Fis binding and minor groove compression

DNA-binding assays using base analogs demonstrate that the guanine 2-amino group is largely responsible for the adverse effects of G/C base pairs on both Fis binding and minor groove compression. As shown in Figure 2A and B, Fis binding to a DNA site (F28–dI) containing three central I/C base pairs (ICI) is increased 60-fold relative to F28 (GCG), to a subnanomolar *K*_d_ closely approaching that of F1 (ATT). Substituting the 5 G/C base pairs in F29 (GGCGC) with I/C base pairs (F29–dI) improves binding 230-fold to within 8-fold of F1. I/C substitutions also increase the lifetimes of Fis–DNA complexes relative to those containing G/C base pairs. Whereas F28 and F29 complexes dissociate almost immediately, half-lives for F28–dI and F29–dI were 14 and 5 min, respectively (Figure 2B).

Fis–DNA complexes containing F28–dI and F29–dI crystallized in the same space group as the canonical Fis–F1 complex and were refined at resolutions of 2.7 and 2.9 Å with an R_w_/R_f_ of 22.1/26.0 and 23.0/27.4, respectively (Table 1). Remarkably, removing only the guanine 2-amino group from the center of the Fis-binding site restores minor groove compression within the Fis complexes to near the widths observed in the F1 complex (Figure 3A–D). The mean and minimum minor groove widths over the central 5 bp are narrowed by 1.3 and 1.0 Å, respectively, in both the F28–dI and F29–dI structures relative to the F28 and F29 structures (Table 2).

### Addition of a purine 2-amino group inhibits Fis binding and minor groove compression

2-aminopurine (2AP), which base pairs with thymine, contains a 2-amino group within the minor groove but is missing the 6-amino group that is normally present in adenine within the major groove (Figure 1C). Fis binding to F28–2AP containing three 2AP/T base pairs within its center is reduced 350-fold relative to the F1 site (Figure 2A and B), a binding penalty that is slightly worse than that observed for F28 with three central G/C base pairs. Like F28, Fis complexes formed with F28–2AP are extremely unstable (Figure 2B).

The co-crystal structure of Fis bound to F28–2AP was refined at 2.8 Å with an R_w_/R_f_ of 21.6/25.9 (Table 1). The minor groove width between positions −2 and +2 is expanded in comparison with the F1 structure and is nearly identical to the minor groove width of F28 (Figure 3A and B). However, the widths of the minor groove opposite to the major groove interfaces (positions −4 to −7 and +4 to +7) in F28–2AP more closely match those of F1 and F28–dI. As with the other structures, the major groove widths and the Fis protein structure are largely unchanged in the F28–2AP complex.

### Effect of the 2-amino group on free-DNA structures studied by molecular dynamics simulations

**Figure 4.**
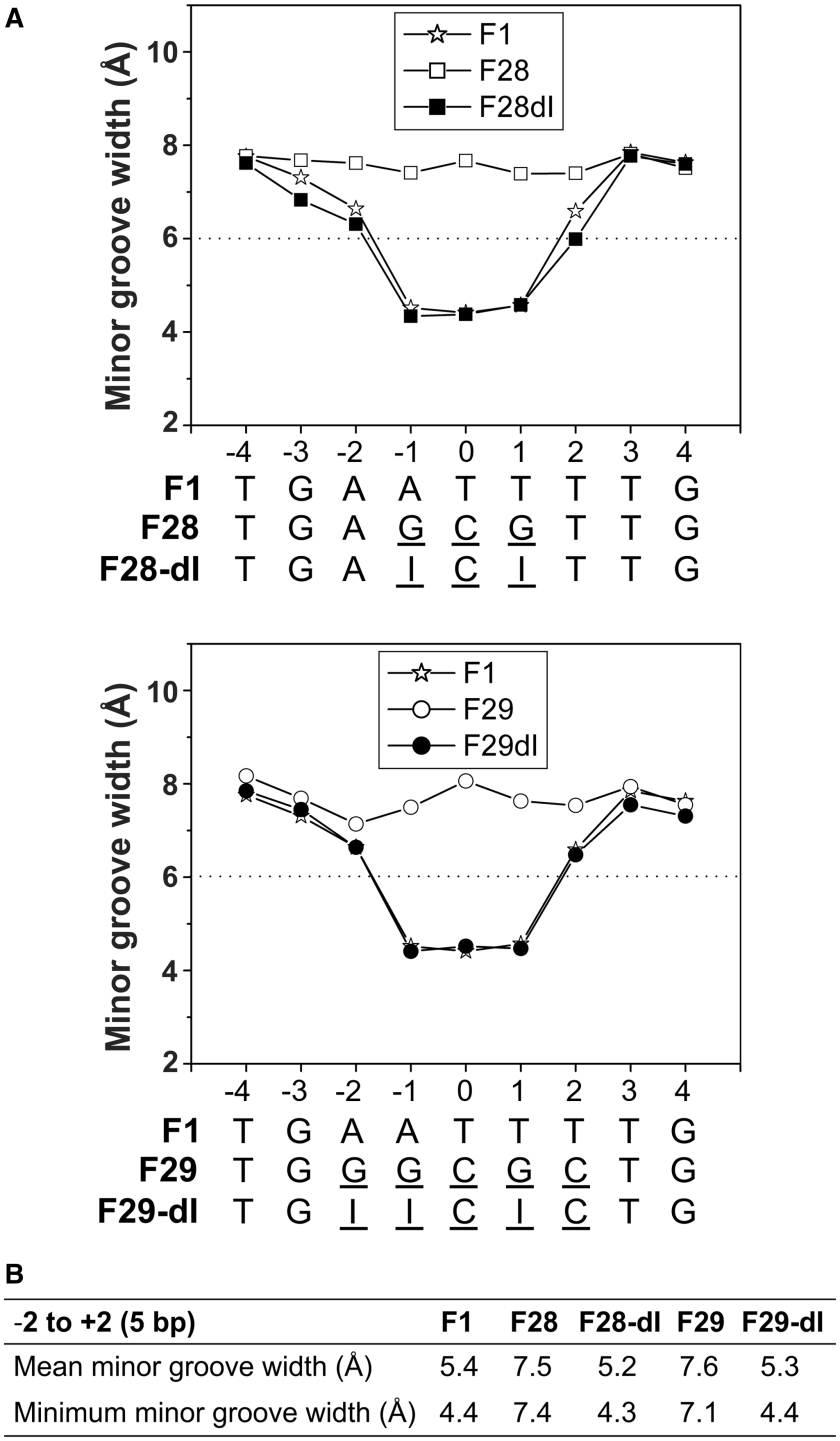
Minor groove widths from 50 ns MD simulations. (**A**) Minor groove width plots over the centers of the F1, F28, F28–dI, F29 and F29–dI sequences. The most probable values along the last 40 ns of the 50 ns MD trajectories are reported (see also Supplementary Figure S3). (**B**) Mean and minimum values for the most probable minor groove widths over the central 5 bp of the simulated sequences.

We performed 50 ns room temperature MD simulations to evaluate the impact of the 2-amino group on minor groove widths of unbound DNA fragments containing the central 15 bp of the F1, F28, F28–dI, F29 and F29–dI sequences. Details on the simulation protocols and statistical analyses are given in the ‘Materials and Methods’ section and Supplementary Figure S3. Statistical analyses of the MD trajectories indicate equilibration as RMSDs (Supplementary Figure S3D), and H-bond lengths (Supplementary Figure S3E) exhibit Gaussian distributions. They also indicate that the most probable value of the minor groove width (Figure 4A) describes the system more accurately than the average value (Supplementary Figure S3A), for each position of each sequence.

In agreement with the experimental data, the MD trajectories reveal that replacement of the guanines with inosines within the central 5 bp of F28 and F29 reduces the width of the minor groove to values that are close to those obtained for the F1 sequence (Figure 4). For F28, the mean and minimum minor groove width over the central 5 bp decreases from 7.5 and 7.4 Å (F28) to 5.2 and 4.3 Å (F28–dI), respectively. Likewise, the mean and minimum minor groove width decreases from 7.6 and 7.1 Å in the F29 structure to 5.3 and 4.4 Å in F29–dI, respectively. Pearson correlation coefficients comparing minor groove width profiles of F28–dI and F29–dI sequences with the F1 sequence are both 0.994, whereas the Pearson correlation coefficient between F28 and F28–dI is 0.525 and between F29 and F29–dI is 0.123. These values demonstrate that the minor groove width profiles of the inosine-containing sequences adopt a shape similar to F1 but significantly different from F28 and F29. We conclude that molecular interactions (e.g. base pairing and stacking) in regions with I/C base pairs are more similar to regions with A/T pairs than to regions with G/C pairs, which result in minor groove shapes of I/C-containing DNA closely mimicking those of A/T-rich DNA.

Interestingly, minor groove widths over the central region exhibit non-Gaussian distributions for F1, F28–dI and F29–dI, whereas the minor groove widths for F28 and F29 and the major groove widths of all five simulated sequences are normally distributed. We also note that there is little effect on major groove widths in the simulations by loss of the 2-amino group (Supplementary Figure S3C).

### Phosphate neutralization and minor groove compression

**Figure 5.**
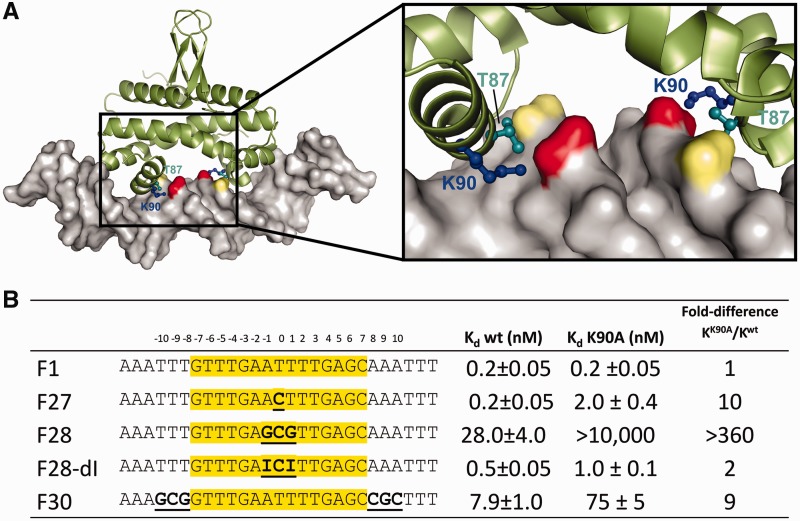
Neutralization of phosphates by Lys90 across the central narrow minor groove facilitates Fis binding depending on the DNA target sequence. (**A**) Structure of the Fis–F1 DNA complex highlighting the side chains of Lys90 (blue) and Thr87 (cyan) that contact the DNA phosphate backbone across the narrowest part of the central minor groove. The separation between Thr87 Cα atoms in the Fis dimer is only 20.1 Å. (**B**) Binding properties of wild-type Fis (wt) and Fis–K90A on different DNA substrates.

In each of the Fis–DNA structures, the side chains of Lys90 extend from the recognition helix D of each subunit to the phosphates spanning the narrowest distance across the minor groove (Figure 5A). Previous studies have reported that substitution of alanine for Lys90 results in only a small effect on Fis binding to some high-affinity sites but strongly reduced binding to lower-affinity sites (25,45,60). Moreover, non-specific DNA binding by Fis–K90A is completely abolished (Supplementary Figure S4A) (25). We show here that binding efficiency of Fis–K90A is directly related to compression of the central minor groove.

Equilibrium binding by Fis–K90A to the optimal F1 sequence (AATTT) is indistinguishable from that of Fis–wt (Figure 5B and Supplementary Figure S4B). However, Fis–K90A binding to a DNA site containing a single G/C substitution (F27; AACTT) is 10-fold worse than Fis–wt, which binds F27 with indistinguishable affinity and structure, including minor groove widths, as for F1 (19). Fis–K90A binding to F28 (AGCGT) is essentially abolished, but binding by Fis–K90A to F28–dI is largely restored (Figure 5B and Supplementary Figure S4B). As a control, we measured Fis binding to the low-affinity binding site F30, which contains three G/C base pairs over the DNA flanking the 15-bp Fis core where the minor groove is also highly compressed (e.g. Figure 1A and B). Fis–K90A exhibited only a 9-fold reduction in binding over Fis–wt to the F30 site (Figure 5B). Taken together, these data demonstrate that the large inhibition of binding observed with Fis–K90A to the F28 and F29 (data not shown) sequences is dominated by the presence of purine 2-amino groups within the center of the binding site. We suggest that neutralization of the proximal phosphates by the salt link with Lys90 is critical for stabilizing the narrow minor groove when sequence features for minor groove compression are suboptimal.

## DISCUSSION

Previous studies have correlated minor groove widths to nucleotide composition, with narrow minor grooves being most often associated with high A/T content (8,33,34). In this article, we show that the exocyclic 2-amino group on guanine is the key determinant responsible for modulating minor groove geometry. MD simulations support this conclusion by showing that free-DNA models of G/C-rich segments lacking the 2-amino group (I/C) generate narrow minor grooves that correspond closely to those of A/T-rich segments. X-ray structures of Fis-bound DNA complexes containing adenine, guanine, inosine and 2-amino purine bases in the non-contacted center of the Fis-binding site reveal that the presence of the purine 2-amino group inhibits minor groove compression. Both X-ray and MD data show that variations in minor groove widths mediated by the purine 2-amino group are not accompanied by corresponding changes in the widths of the major groove.

Narrowing of the minor groove is directly correlated with Fis protein binding. The presence of 2-amino groups over the central 5 bp of the Fis-binding site (F29) inhibits binding 225-fold relative to the identical DNA sequence lacking 2-amino groups (F29–dI). Likewise, the presence of 2-amino groups over only the central 3 bp of the Fis recognition site inhibits binding 56- to 140-fold (F28 or F28-2AP compared with F28–dI). In each of these cases, Fis complexes formed with DNAs containing 2-amino groups show less compressed minor grooves over shorter DNA regions than those formed with A/T or I/C base pairs. Not surprisingly, all complexes containing purine 2-amino groups are extremely unstable. To our knowledge, this is the first direct demonstration of the impact of the 2-amino group on minor groove width that is supported by X-ray crystal structures, computational prediction and functional protein binding.

An intrinsically narrow or dynamically compressible minor groove is required for Fis binding because of the unusually short separation between recognition helices in the dimer. Because the protein structure does not change upon DNA binding, even when binding to a poor site, the DNA structure must conform to the shape of the Fis-binding surface. Our MD simulations, together with surveys of free-DNA X-ray structures (8), provide evidence that high-affinity Fis-binding sites containing A/T-rich centers have intrinsic minor groove shapes that resemble the bound conformation. On the other hand, Fis is able to bind to many suboptimal binding sites, even random sequence DNA, albeit at severe energetic costs and with short lifetimes when compared with optimal sequences. Although such sequences are not expected to typically exhibit narrow minor groove regions, we propose that the dynamic nature of DNA structure enables Fis to select transiently narrow minor groove segments (Supplementary Figure S3B), which can then be stabilized through Coulombic interactions with the electropositive binding surface of the protein.

Studies on several other DNA-binding proteins that bind in a more sequence-specific fashion than Fis have also revealed a critical importance of minor groove shape. Two well-studied examples are the phage 434 and P22 c2 repressors whose binding site centers also contain A/T base pairs and whose dimeric HTH motifs are spaced closer than the average pitch of B-DNA (17,61). Unlike Fis, however, the 434 repressor inserts arginines into the narrow and, hence, more electronegative minor groove. The human papillomavirus E2 transactivators are examples of non-HTH DNA-binding proteins that require a central compressed minor groove (18). Binding of E2 is also accompanied by significant bending within the binding site center, whereas the centers of the Fis-, 434 repressor- and P22 c2 repressor-binding sites are nearly straight. In all cases, G/C base pairs introduced into the central compressed minor groove region strongly inhibit binding (20,62,63). For the 434 repressor, this inhibition has also been correlated to the purine 2-amino group by binding studies with base analogs (64).

### How does the 2-amino group control minor groove widths?

Previous structural modeling of Fis-binding sites with A/T-rich centers has shown that small amounts of negative roll, overtwisting and negative slide values that are present in the crystal structures are uniquely sufficient to reconstruct DNA molecules that closely fit the conformations in the complexes (e.g. F1 in Supplementary Figure S5) (19). This is also true for the structures containing inosines reported here (Supplementary Figure S5). Helix twist, roll and slide values alone are less able to reproduce the structure in the crystal for the DNAs containing purine 2-amino groups, particularly in the case of F29 containing five G/C base pairs (Supplementary Figure S5). Of these three DNA parameters, helix twist shows the greatest deviations in the X-ray structures of Fis–DNA complexes containing 2-amino groups (Supplementary Figure S2), as their minor grooves are forced to adopt a geometry that is compressed sufficiently to fit the Fis-binding surface. Roll is less affected but exhibits overall less negative values over the centers of the F28, F29 and F28–2AP structures relative to those that lack the 2-amino group (F1, F28–dI and F29–dI) (Table 2 and Supplementary Figure S2). Thus, the 2-amino group may restrict variability in helix twist and roll that facilitate minor groove compression. We note that large negative propeller twists and bifurcated hydrogen bonding, which are often characteristic of A-tract DNA (32), do not seem to correlate with minor groove compression in the Fis structures (Table 2 and Supplementary Figure S2).

To provide additional insights into the role of the purine 2-amino group in restricting minor groove compression, we built DNA models of the F28 (GCG) sequence with the F28–dI or F1 (ATT) helical parameters using 3DNA (49). These models contain minor grooves optimally compressed for Fis binding. However, all three of the purine 2-amino groups in these models are involved in steric clashes, mostly with other exocyclic groups associated with paired or diagonally apposed bases (Supplementary Figure S6).

An additional factor that could contribute to the effect of the purine 2-amino group on minor groove shape is the distribution of waters and cations within the groove (37,42,65). However, comparison of high-resolution crystal structures of decamers with identical sequence, except for the central 2 bp being I/C (PDB ID: 1D61) or G/C (PDB ID: 5DNB), shows only small differences in the first hydration shell and little difference on groove widths (44,66). Recent high-resolution structures comparing the P22 c2 repressor bound to ATAT with ACGT again show only small hydration differences, with the waters directly over the CG 2-amino groups being more mobile, and no evidence for ordered cations within the narrow minor groove (20). Nuclear magnetic resonance using ^23^Na and microsecond MD simulations have provided evidence for localized cation binding over the AATT sequence in the Dickerson dodecamer (67,68). The same A-tract is present at the center of the F1 sequence, and our I/C-substituted DNAs may have a similar potential for ion binding. However, as these studies found that the ion-binding site is occupied only a few percent of the time, both studies concluded that ion binding could not be a major determinant responsible for minor groove narrowing. Although our Fis–DNA structures are not of sufficient resolution to draw conclusions about water or ion content, we do not believe that the current evidence is compelling for a primary role in controlling minor groove widths. Instead, we suggest that intrinsic structural properties of DNA governed by the purine 2-amino groups are most important for determining minor groove geometry. Nevertheless, hydrogen bonding networks involving waters and ions within the groove may contribute to stabilization of narrow minor grooves (32,37,40,68).

### Protein-mediated neutralization of phosphates can contribute to minor groove narrowing

We find that lysine contacts to phosphates across the narrowest part of the central minor groove in the Fis complex are critical for binding in certain sequence contexts. Thus, unlike wild-type Fis, Fis–K90A is unable to form a complex when three G/C base pairs are present in the center of the Fis-binding site, and it even shows a 10-fold effect on equilibrium binding when a single G/C base pair is present at the binding site center. On the other hand, Fis–K90A exhibits near wild-type binding to sites with A/T or I/C centers. These data lead us to suggest that neutralization of the proximal phosphates by the salt link with Lys90 is a mechanism for stabilizing the narrow minor groove when sequence features for minor groove compression are suboptimal.

A role for phosphate charge neutralization in controlling minor groove geometry is supported by earlier MD simulations where partial neutralization of six phosphates spanning a stretch of four G/C base pairs resulted in a 2.5 Å decrease in the minimum minor groove width (69). Similar phosphate charge neutralization over an already narrowed A/T region caused only a small reduction (∼1 Å) in the minimum groove width.

### Binding site selection and Fis–DNA complex assembly

Our data support a model whereby Fis initially selects binding sites containing an intrinsically narrow minor groove that is primarily controlled by the presence of purine 2-amino groups. Lys90 on Fis may be particularly important in the initial scanning step by facilitating Fis-induced narrowing of sequences that contain some G/C base pairs. A DNA segment with a narrow minor groove enables the recognition helices from each subunit of the dimer to insert into the adjacent major grooves. Modest bends within the major groove interfaces enable DNA backbone contacts, and for high-affinity sites, formation of arginine–guanine bidentate hydrogen bonds at each end that further mold the DNA onto the Fis protein surface. The assembled complex contains radical variations in minor groove shape throughout the binding interface and considerable overall DNA bending (65° in the crystal structures), but the shape of the major groove remains relatively constant.

## ACCESSION NUMBERS

4IHV, 4IHW, 4IHX and 4IHY.

## SUPPLEMENTARY DATA

Supplementary Data are available at NAR Online: Supplementary Figures 1–6 and Supplementary Reference [70].

## FUNDING

National Institutes of Health (NIH) [GM038509 to R.C.J.]; Italian Institute of Technology [project MOPROSURF and computational platform to R.D.F.]; Alfred P. Sloan Research Fellowship (R.R.). The UCLA-DOE X-ray Crystallography Core Facility is supported by the Department of Energy [E-FC02-02ER63421]. The Advanced Photon Source (APS) beamlines 24-ID are supported by the National Center for Research Resources [5P41RR015301-10]; National Institutes of General Medical Sciences [8 P41 GM103403-10]. Use of the APS is supported by the Department of Energy [Contract DE-AC02-06CH11357]. Funding for open access charge: NIH.

*Conflict of interest statement.* None declared.

## Supplementary Material

Supplementary Data
