# Supplementary material for: Control of DNA minor groove width and Fis protein binding by the purine 2-amino group
Source: Nucleic Acids Res. 2013 May 9;41(13):6750–60. doi: 10.1093/nar/gkt357 (PMC3711457; doi:10.1093/nar/gkt357)
Supplement: Supplementary Data [file supp_41_13_6750__index.html]

Control of DNA minor groove width and Fis protein binding by the purine 2-amino group — Control of DNA minor groove width and Fis protein binding by the purine 2-amino group — Supplementary Data 

# Control of DNA minor groove width and Fis protein binding by the purine 2-amino group

## Supplementary Data

files

**Files in this Data Supplement:**

- Supplementary Data - pdf file
