# Supplementary material for: Control of DNA minor groove width and Fis protein binding by the purine 2-amino group
Source: Nucleic Acids Res. 2013 May 9;41(13):6750–60. doi: 10.1093/nar/gkt357 (PMC3711457; doi:10.1093/nar/gkt357)
Supplement: Supplementary Data [file supp_gkt357_nar-00699-f-2013-File009.pdf]

Supplementary Figures for:

Control of DNA Minor Groove Width and Fis Protein Binding by the Purine 2-amino Group

Stephen P. Hancock, Tahereh Ghane, Duilio Cascio, Remo Rohs, Rosa Di Felice & Reid C. Johnson

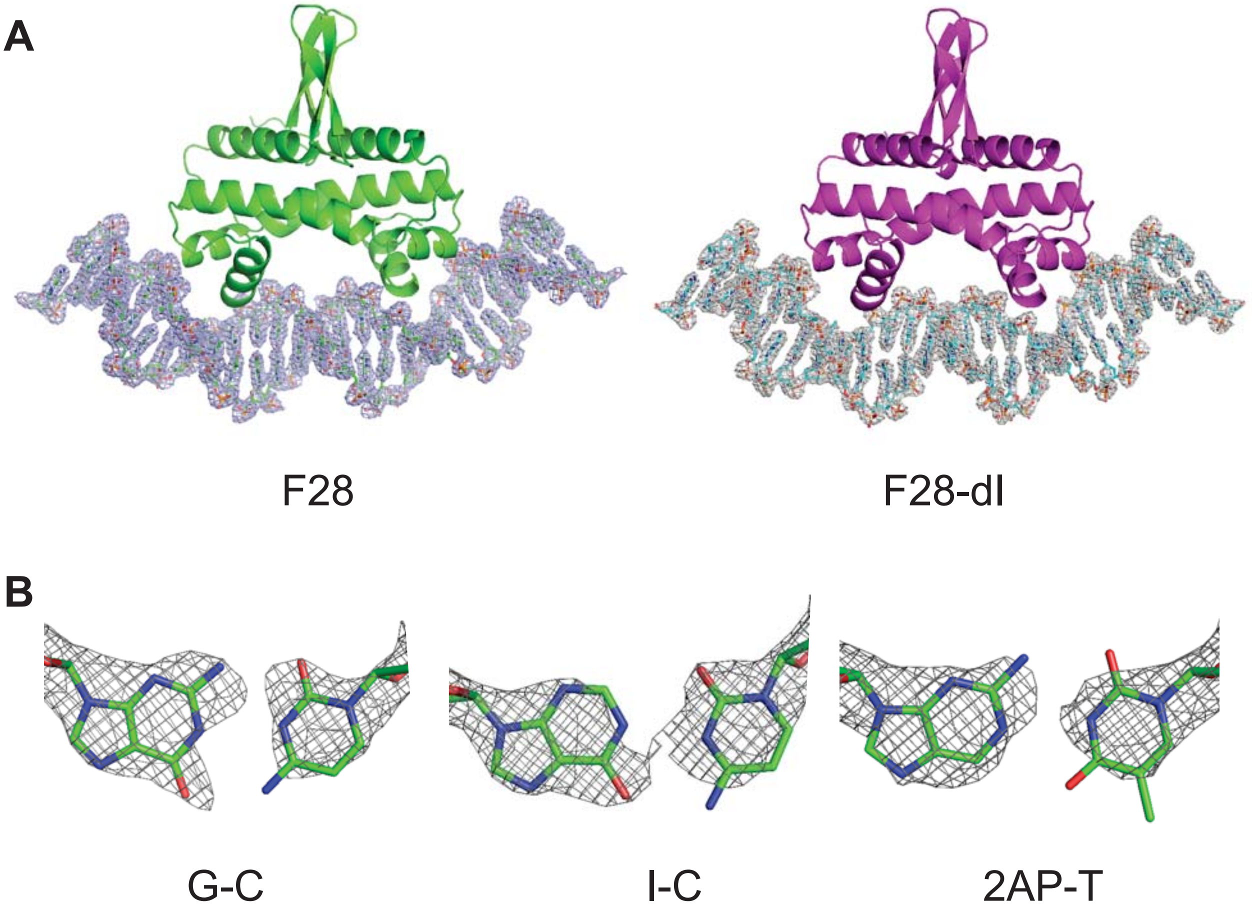

Figure S1. Representative electron density maps.
(**A**) 2Fo-Fc electron density maps for the DNA in the F28 (GCG) and F28-dI (ICI) models contoured at 1.5 σ.
(**B**) 2Fo-Fc electron density maps contoured at 2.0 σ of representative base pairs containing modified bases. Each purine is the +1 base from the F28, F28-dI or F28-2AP crystal structures.

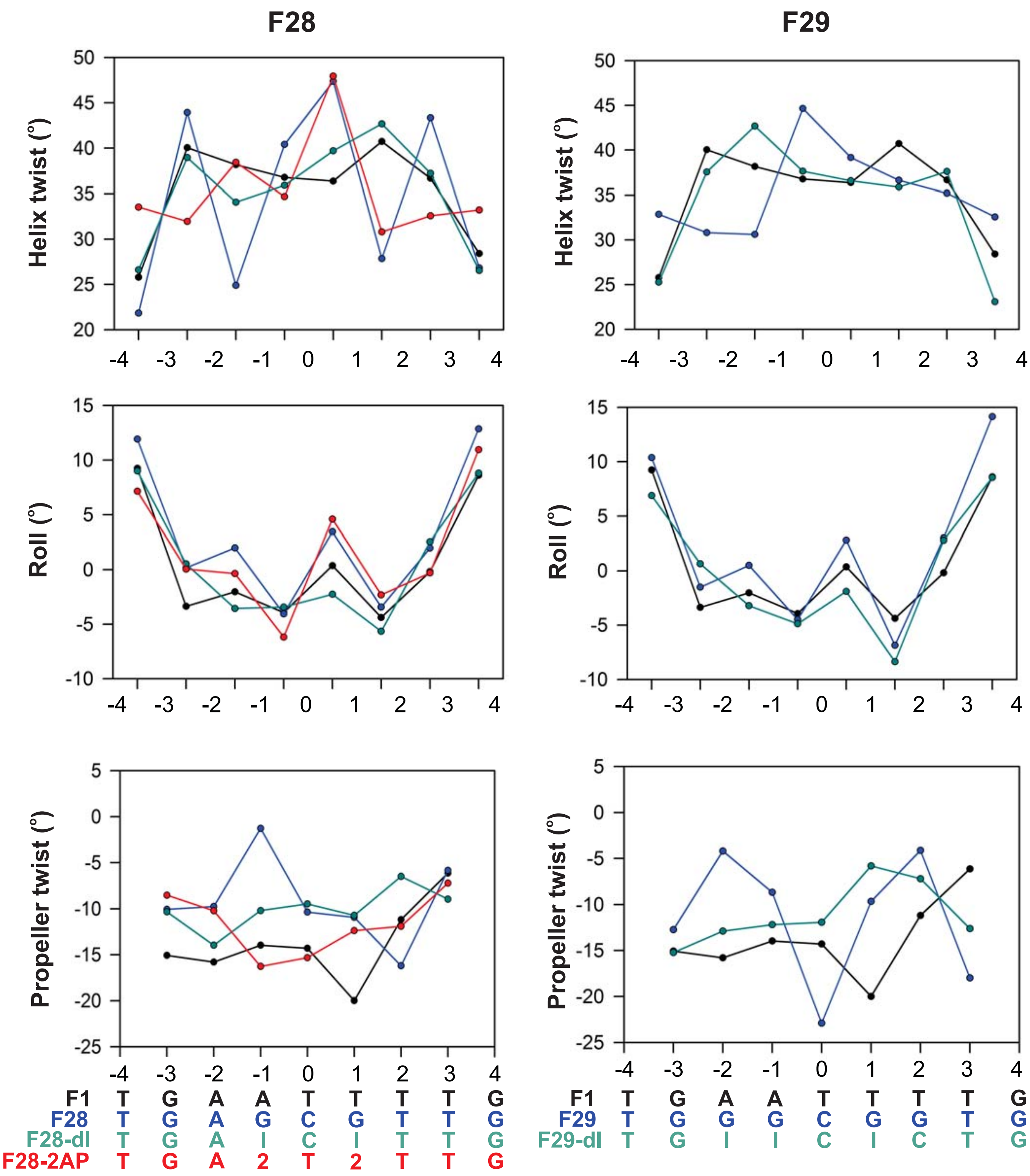

Figure S2. Helix twist, roll, and propeller twist parameters across the centers of the Fis-DNA X-ray structures. The F28 and F29 sets of DNA structures are on the left and right panels, respectively. Color coding follows the DNA sequences given on the bottom.

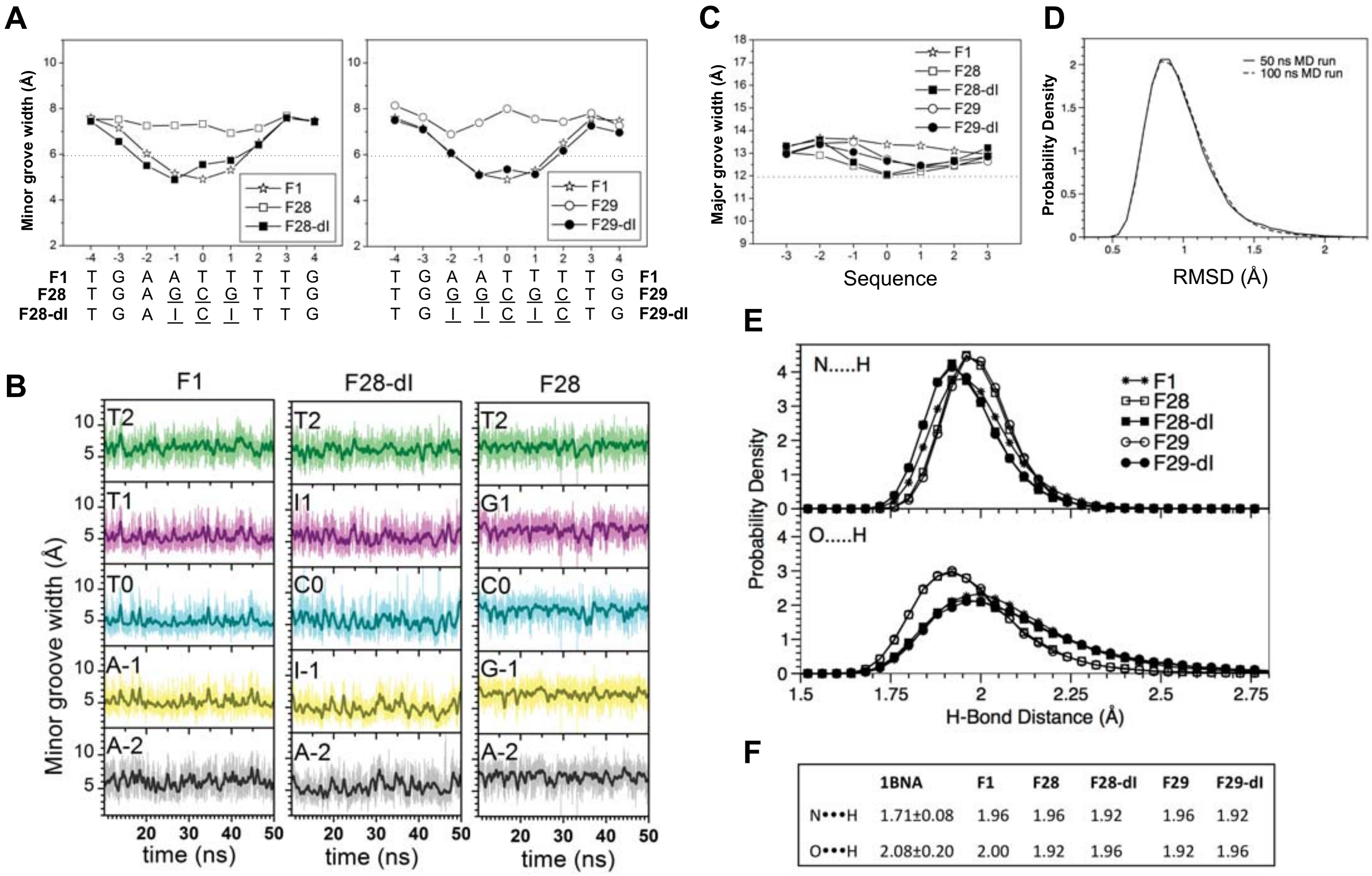

Figure S3. Time behavior and statistics of the MD trajectories.
(**A**) Minor groove widths over the central regions of the F1, F28, F28-dI, F29, and F29-dI sequences. The average values along the last 40 ns of the trajectories are reported (compare with the most probable values plotted in Figure 3). The horizontal dotted lines mark the canonical minor groove width for B-DNA. The average minor groove width values also demonstrate that I/C base pairs are more similar to A/T base pairs than to G/C base pairs.
The overestimation of the minor groove width by MD simulations was previously noted (70). Important for this study is the accurate description of the sequence-dependent patterns, which support the conclusion that the decrease in minor groove width by G to I substitution is a genuine result of the dynamics. We also note large fluctuations (70): the complex may remain trapped in configurations with particularly narrow minor grooves among those sampled during the dynamics at finite temperature.
(**B**) Minor groove width as a function of time for representative sequences; similar behaviors are observed for F29 and F29-dI. Light curves: bare data from MD. Dark curves: smoothing over 0.5 ns has been applied. Different colors (gray, yellow, cyan, magenta, green) are used for the different nucleotide locations (from -2 to +2, respectively).
(**C**) Major groove widths over the central regions of the F1, F28, F28-dI, F29, and F29-dI sequences. The average values along the last 40 ns of the trajectories are reported. The horizontal dotted line marks the canonical major groove width for B-DNA. Negligible changes of the major groove width emerge by changing the sequence, in contrast to the changes of the minor groove width.
(**D**) Probability density of the root mean square deviation (RMSD) for F1 over the heavy atoms in the central 7 bp segment from the 50 ns trajectory and from a test 100 ns trajectory. The very good agreement indicates that our 50 ns trajectories are sufficiently long to capture the overall behavior of this system.
(**E**) Statistics of the H-bond distances in the central base pair (AT in F1, GC in the other oligomers): the plots represent the probability density of two different kinds of H-bonds (top and bottom) for the various sequences. The distributions are normally peaked around the most probable (average) value, which is a good indication of reliable trajectories.
(**F**) Average values of the H-bond lengths in the central base pair from our simulations, compared to those in AT pairs in the central portion of the Dickerson dodecamer (PDB ID: 1BNA).

70. Pérez, A., Lankas, F., Luque, F.J. and Orozco, M. (2008) Towards a molecular dynamics consensus view of B-DNA flexibility. *Nucleic Acids Res.*, **36**, 2379-2394.

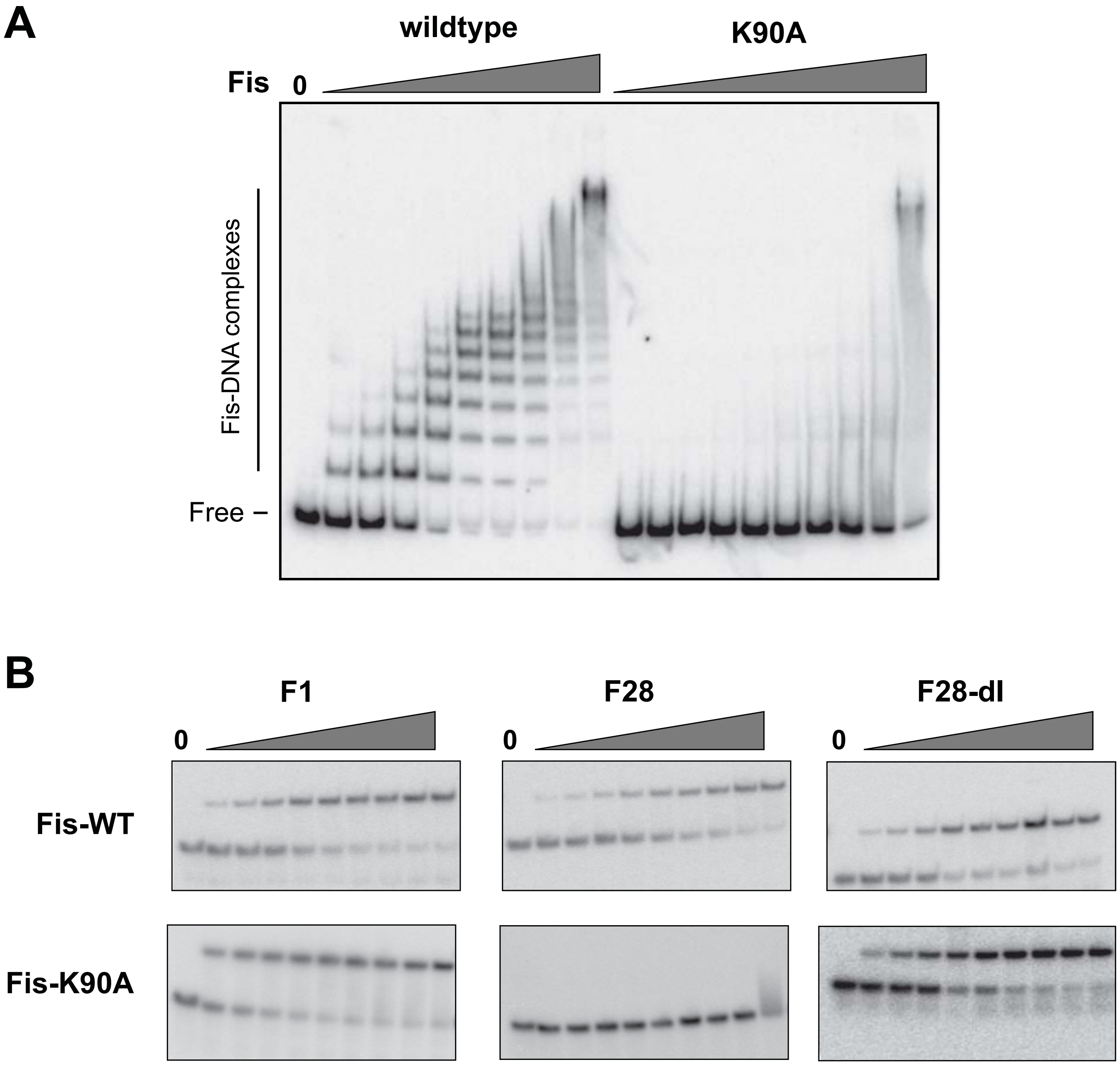

Figure S4. Binding of Fis-K90A to non-specific and specific DNA substrates.
(**A**) Gel mobility shift assays performed without competitor DNA measuring non-specific binding of Fis-WT and K90A on a 149 bp actin gene fragment from *S. cerevisiae*. Fis-WT was added in 2-fold increasing concentrations from 0.5 to 128 nM and Fis-K90A from 0.5 to 256 nM.
(**B**) Representative gel mobility shift assays (+50 μg/ml poly dI-dC) on 27 bp F1, F28 and F28-dI DNA duplexes. Fis-WT was added in increasing concentrations from 0.05 to 20 nM, 2.5 nM to 1 μM, and 0.1 to 40 nM to F1, F28 and F28-dI, respectively, and Fis-K90A from 1 to 400 nM, 200 nM to 8 μM and 0.25 to 100 nM for F1, F28 and F28-dI, respectively.

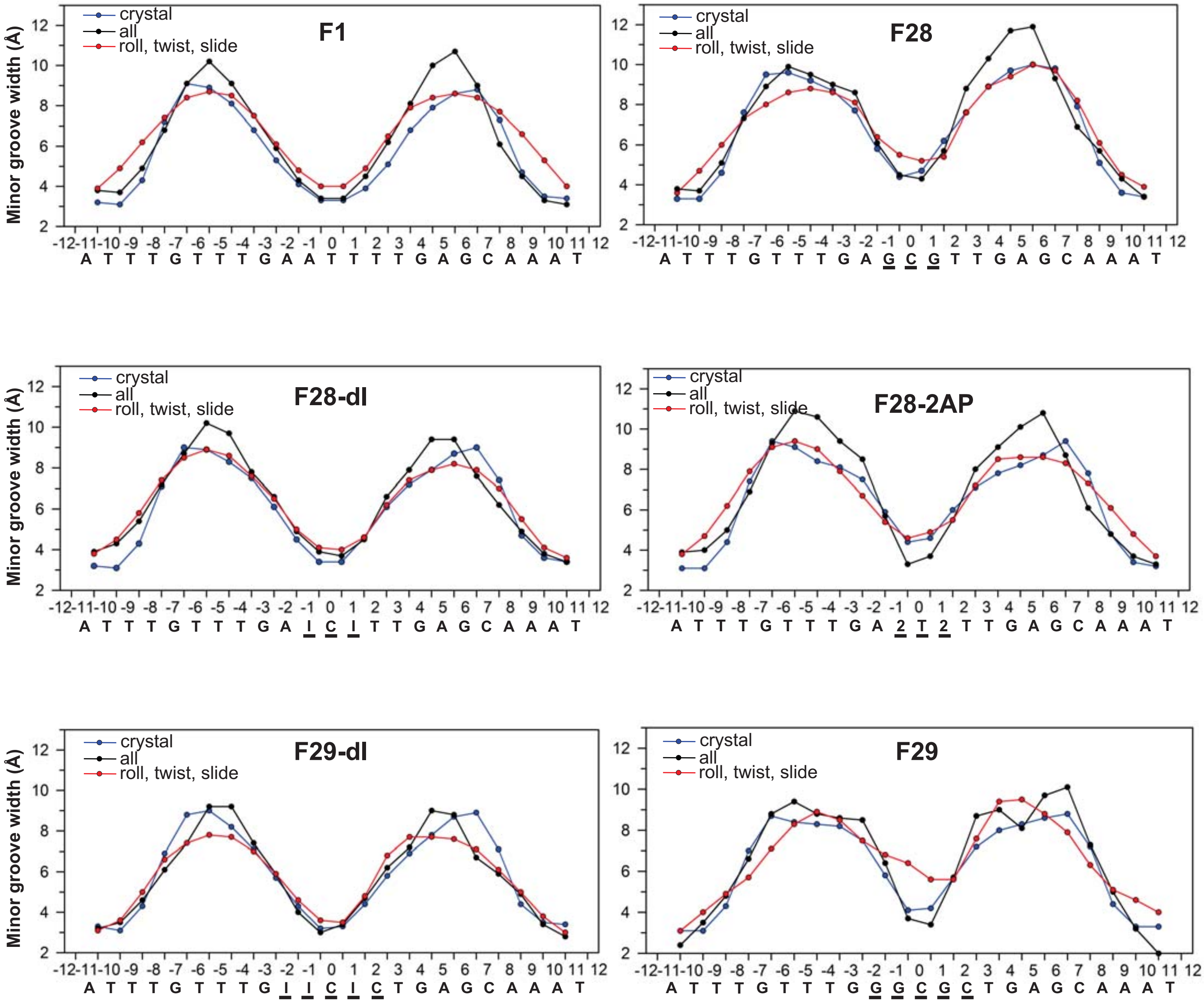

Figure S5. Minor groove plots comparing DNA structures in the Fis-DNA crystals with those of DNA molecules rebuilt *in silico* using parameters derived from X-ray structures.

For all plots blue points/lines represent the minor groove widths in the crystal structure; black points/lines represent DNA molecules reconstructed using all 12 base pair and base pair step parameters from the crystal structure of the same sequence; red points/lines represent DNA molecules constructed with only the roll, helix twist, and slide parameters from the crystal structure and mean sequence-specific base pair and base-pair step values (Olson et al., 1998) of the same sequence. The sequence is listed at the bottom of each panel. Note that the minor groove widths of F1 and the inosine-containing structures built with only the roll, helix twist, and slide parameters relatively closely match those of the X-ray structures or those rebuilt from all 12 parameters, but the DNA structures with the purine 2-amino group (F28, F28-2AP and F29) exhibit poorer matches.

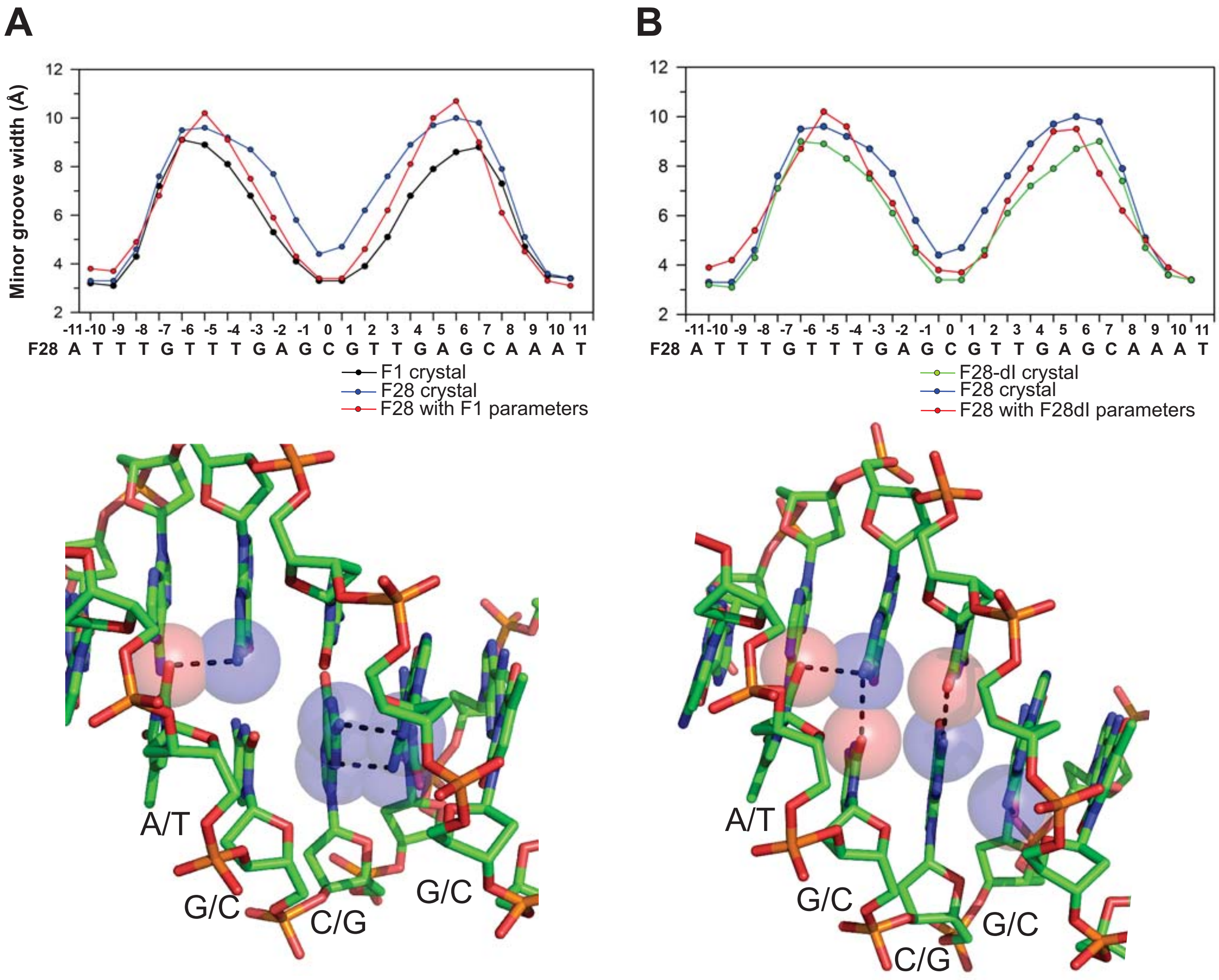

Figure S6. DNA models of the F28 (GCG) sequence constructed using parameters from: (**A**) the F1 (ATT) crystal structure or (**B**) the F28-dI (ICI) crystal structure.

Minor groove plots of the models along with the relevant crystal structures are on the top, and the model structures highlighting clashes involving the guanine 2-amino groups are on the bottom. In the F28 sequence model based on F1 complex parameters (A), the central guanine 2-amino groups clash against diagonally-apposed bases (thymine O2 or guanine N1), whereas in the model based on F28-dI complex parameters (B), the clashes are against directly-apposing cytosine O2 atoms and against a diagonally-apposed thymine O2 atom.
